# Impact of various storage media and time on mechanical properties of bovine root dentin

**DOI:** 10.1038/s41598-026-47214-1

**Published:** 2026-04-11

**Authors:** Julia Herzog, Merle Louisa Klümke, Bogna Stawarczyk, Melisa Klimenta, Philipp Kanzow, Tina Rödig, Franziska Haupt

**Affiliations:** 1 Department of Preventive Dentistry, Periodontology and Cariology, University Medical Center Göttingen, Robert-Koch-Str. 40, 37075 Göttingen, Germany; 2 Department of Prosthetic Dentistry, University Hospital LMU Munich, Goethestraße 70, 80336 Munich, Germany; 3 Department of Restorative Dentistry, Periodontology and Endodontology, University Medicine Greifswald, Walther-Rathenau-Straße 42a, 17475 Greifswald, Germany

**Keywords:** Storage solution, Martens hardness, Microindentation, Dentin, Thymol, Chloramine-T, Biomaterials, Dentine

## Abstract

In-vitro-studies usually require extracted teeth, which have been stored over an extended period of time. It can be assumed that storage solutions and time may alter the mechanical properties of dentin. The aim of this study was to examine Martens hardness parameters of bovine dentin with regard to the storage medium and storage time for up to 6 months. Standardized root dentin bars from extracted bovine incisors were stored in thymol 0.1%, chloramine-T 0.5%, formalin 10% and double distilled water. At baseline and after storage periods of 1, 3, and 6 months, samples were analyzed regarding Martens hardness, indentation hardness, indentation modulus, and indentation creep (linear mixed-effects regression models). Within 3 months of storage at the latest, all solutions showed significantly reduced values for Martens and indentation hardness. Regarding the indentation modulus, a reduction was observed for all groups at 6 months at the latest. In general, chloramine-T was shown to have the least impact on the mechanical properties of bovine dentin. Within the limitations of this study, storage medium and time should always be considered when conducting studies on the mechanical properties of bovine dentin, including any unfavorable changes of the dental hard tissues.

**Supplementary Information:**

The online version contains supplementary material available at 10.1038/s41598-026-47214-1.

## Introduction

Dental in-vitro studies evaluating the mechanical properties and microstructural alterations of human or bovine dentin mainly use extracted teeth^1,2^. In general, storage of collected teeth in liquids is inevitable as dry storage leads to increased stiffness and brittleness of dental hard tissues^3^. Normally, teeth are stored in distilled water or aqueous solutions to which disinfecting agents such as chloramine-T or thymol have been added. Although storage in aqueous solutions preserves hydration, it may also affect the mineral content of dentin resulting in changes of the mechanical properties^4,5^. Therefore, extracted teeth should be properly stored to obtain reliable measurements^2^. It was shown previously that storage in distilled water or disinfecting agents such as formalin or thymol may alter the mechanical properties of mineralized hard tissues^6–8^. Assessment of the flexural, tensile or compressive strength as well as the fracture resistance are commonly used to evaluate mechanical properties of tooth substances^9–12^. However, these techniques are associated with several drawbacks as neither local analysis of a specific structure, nor the evaluation of the whole specimen is possible. Furthermore, these analyses often require destruction of the tooth^10,11^ which impedes repeated measurements over a specific period of time. Alternatively, instrumented indentation tests with measurements in a nano- or micrometer range were applied^13–15^.

The instrumented indentation method is carried out by pressing an indenter—typically a diamond with a known geometry—into the surface to be examined. During the indentation process, both the indentation depth and the load are continuously recorded throughout the insertion and withdrawal of the indenter. Thereby, a loading and unloading curve is generated that depicts the applied load as a function of the indentation depth. Consequently, the instrumented indentation technique can assess the Martens hardness which accounts for both plastic and elastic deformation^15^. An alternative technique to determine mechanical properties is a static indentation test assessing the Vickers hardness which requires the visualization and measurement of indents and consider only the permanent (plastic) deformation^16,17^. For that reason, these techniques do not provide detailed information about the materials behavior under load. Till today, the knowledge about the optimal storage solution for dental hard tissue is scarce and studies are hardly comparable due to different media, methodologies and storage periods^6,7,18–20^. Furthermore, there are only few studies evaluating the effect of disinfecting agents on dentin after storage for several months^6,18^.

Therefore, the aim of the present study was to compare the effect of different storage media on the mechanical properties of bovine root dentin for a storage period of up to 6 months.

## Methods

Specimens were obtained from calves’ teeth that were collected as waste products during the slaughter process in slaughterhouses. Only bovine central incisors without carious lesions and fully developed roots were selected and included in the study. All teeth were stored in a glass container with distilled water at room temperature until use to prevent dehydration with a maximum storage duration of 4 months. To prepare root dentin bars, all teeth were embedded with their crowns in acrylic resin (PalaXpress ultra, Kulzer, Hanau, Germany) to ensure stability during sectioning. Forty identical bars of 10 mm × 4 mm × 1.2 mm size were sectioned with a precision cutting machine using a water-cooled diamond cut-off wheel (Secotom-50 with M1D13, Struers, Willich, Germany) at a speed of 3300 rpm. All dentin bars were coated with Vaseline prior to embedding in acrylic resin (SCANDIQUICK, Scan-Dia, Hagen, Germany) to prevent resin infiltration into the dentin tubules on the measuring surface. Then, the measuring surface of each embedded bar was polished with 500-grit sandpaper to remove any residual resin as required for the testing machinery. The 500-grit sandpaper was used consistent to previous studies reporting a range between 320-grit and 4000-grit^6,15,18^. All dentin bars were prepared by one operator.

The following solutions served as storage media (n = 10 each): double distilled water (Kerndl, Vaterstetten, Germany), chloramine-T 0.5% (Carl Roth, Karlsruhe, Germany), thymol 0.1% (Caesar & Loretz, Hilden, Germany), formalin 10% (Carl Roth). When multiple samples (up to three) were obtained from a single tooth, they were intentionally assigned to different media to minimize potential bias in the results. The sample size of ten specimens per group was based on three studies evaluating the impact of storage duration on the mechanical behavior of dentin while using indentation techniques^6,7,20^. Prepared samples were stored in the respective solutions in resealable white glass containers at an ambient temperature of 8 °C in a controlled and light-protected cooling device. Compared to a previously published study, liquids were not replenished during the 6 months of storage^6^.

After removing excess liquid on the surface with absorbent paper, samples were subjected to instrumented indentation testing using a universal testing machine calibrated to manufacturer’s instructions (ZHU 0.2, ZwickRoell, Ulm, Germany) and mounted with a Vickers diamond indenter (α = 136°). Load–displacement curves were recorded force-controlled. All surfaces were loaded with a maximum of 9.81 N. After achieving contact with the tested surface, the load was applied with a velocity of 0.5 mm/min, followed by a dwell time of the test force of 10 s and a removing speed of 0.1 mm/min. Each measurement included three indentations per specimen at differently placed points, which were determined by using the in-built measuring microscope (inherent magnification 40×, camera resolution: 1.4 megapixel). Throughout the testing process of about 2 min special attention was paid to ensure the dentin remained hydrated.

Martens hardness (*HM*, N/mm^2^), indentation hardness (*H*_*IT*_, N/mm^2^), indentation modulus (*E*_*IT*_*,* kN/mm^2^), and indentation creep (*C*_*IT*_, %) were determined (testXpert v.12.3, ZwickRoell) using the formulas given in the ISO 14577-1 specification^21^.

The *HM* is defined as the quotient of the maximum load *F*_*max*_ and the corresponding contact area *A* of the indenter at the time of the maximum load.

$$HM=\frac{{F}_{max}}{{A}_{s}(h)}=\frac{{F}_{max}}{\mathrm{26,43}{h}^{2}}$$

with *HM* in N/mm^2^, *F*_*max*_ (maximum test force) in N, *A*_*s*_* (h)* (surface area of the indenter at distance *h* from the tip) in mm^2^, and *h* (indentation depth under applied test force) in mm.

While *HM* depends on plastic and elastic material properties, *H*_*IT*_ relates to the resistance against plastic deformation.

$${H}_{IT}=\frac{{F}_{max}}{{A}_{p}}=\frac{{F}_{max}}{\mathrm{24,43}{h}_{c}^{2}}$$

with *H*_*IT*_ in N/mm^2^, *A*_*p*_ (projected [cross-sectional] area of contact between the indenter and the test piece) and *h*_*c*_ (indentation depth due to plastic deformation).

The indentation modulus was calculated as follows:

$${E}_{IT}=\left(1-{v}_{s}^{2}\right) x {(\frac{1}{{E}_{r}}-\frac{(1-{v}_{i}^{2})}{{E}_{i}} )}^{-1}\text{ with }{E}_{r}=\frac{\sqrt{\pi }}{2C\sqrt{{A}_{p}}}$$

with *E*_*IT*_ in kN/mm^2^, *E*_*r*_ (reduced modulus of the indentation contact) in N/mm^2^, *E*_*i*_ (elastic modulus of the indenter) in N/mm^2^, *C* (compliance of the contact), *v*_*s*_ (Poisson ratio of the test piece) = 0.35 and *v*_*i*_ (Poisson ratio of the indenter) = 0.07^22,23^.

The force-controlled measurements allow for determining the material’s creep (*C*_*IT*_), which is defined as the change of indentation depth under constant load in percent: by the increase in the indentation depth starting from an indentation depth *h*_1_ to an increased depth *h*_2_ after applying the maximum test force. Therefore, the relevant parameters were calculated as follows:

$${C}_{IT}=\frac{{h}_{2}-{h}_{1}}{{h}_{1}}100\%$$

with *h*_1_ (indentation depth when reaching *F*_*max*_) in mm and *h*_2_ (indentation depth at the end of dwell time of *F*_*max*_) in mm.

Measurements were carried out at the following time points: T0 (before storage), T1, T3 and T6 (storage periods of 1, 3, and 6 months). Per specimen three indentation measurements were taken at different surface points. For statistical analysis, these values were treated as three separate data entries.

Calculations were carried out using the software R (www.r-project.org, version 4.2.1) and the packages “lme4” (version 1.1–34), “afex” (1.3.0), and “emmeans” (version 1.8.7). Linear mixed-effects regression models were used to assess potential effects for each storage media over time (T1, T3, and T6) compared to T0. The storage media and the interaction between storage media and storage duration were entered as fixed effects. Repeated measures (i.e. assessment after different storage durations) were considered by modelling random intercepts and random slopes per specimen and per tooth. For the comparison between different storage media at each time point, pairwise comparisons based on estimated marginal means were performed. The level of statistical significance was set at α = 0.05. Resulting *p* values from pairwise comparisons were adjusted, and the multivariate *t* distribution was used to assess the probability/critical value.

## Results

**Fig. 1:**
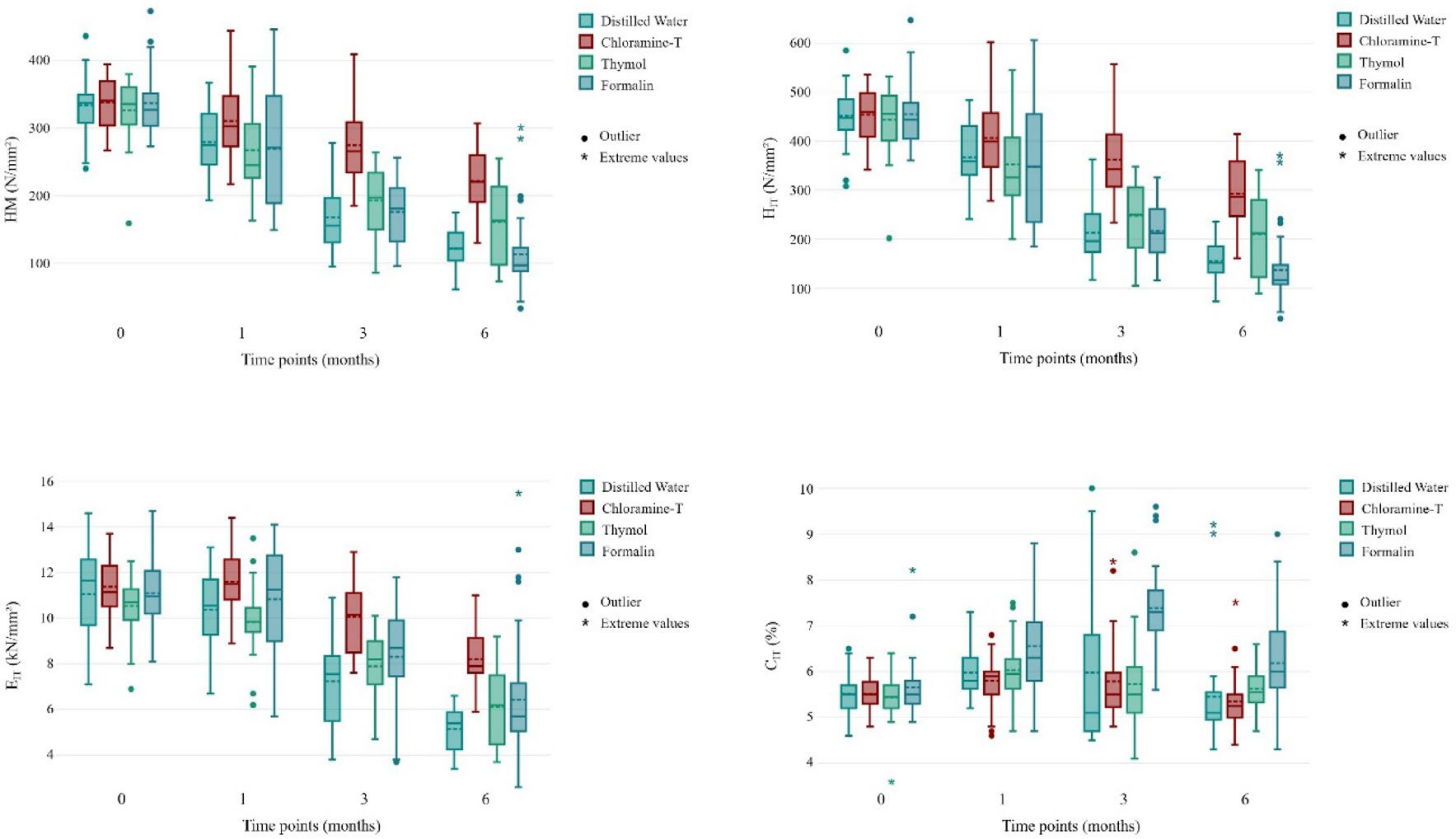
Boxplots showing the hardness parameters HM (Martens hardness; N/mm²), H_IT_ (indentation hardness; N/mm²), E_IT_ (indentation modulus; kN/mm²) and C_IT_ (indentation creep; %) for all time points (T0, T1, T3, T6).

Characteristics of baseline data (T0) and subsequent measurements after 1, 3 and 6 months (T1, T3, T6) are shown for each parameter in Table 1, Supplemental Tables 1–4, and Fig. 1. There were no significant differences between groups at the baseline measurements for all parameters.

**Table 1:** Depiction of median and mean values including standard deviation of hardness parameters HM (Martens hardness; N/mm^2^), H_IT_ (indentation hardness; N/mm^2^), E_IT_indentation modulus; kN/mm^2^) and C_IT_ (indentation creep)

Time point	T0		T1			T3			T6		
Medium	Median	Mean (± SD)	Median	Mean (± SD)	∆T1–T0 (%)	Median	Mean (± SD)	∆T3–T0 (%)	Median	Mean (± SD)	∆T6–T0 (%)
Martens hardness (N/mm^2^)											
Distilled water	337.00	333.73 (43.62)	275.00	279.30 (46.59)	− 16.31	155.50	167.70 (50.42)	− 49.75	119.00	121.50 (27.93)	− 63.59
Chloramine-T	340.50	338.13 (37.02)	302.50	310.20 (53.18)	− 8.26	265.50	274.77 (59.82)	− 18.74	220.50	222.07 (50.73)	− 34.33
Thymol	335.50	326.30 (45.97)	245.00	267.17 (59.51)	− 18.12	197.00	192.72 (49.34)	− 40.94	163.00	160.80 (58.77)	− 50.72
Formalin	327.00	336.80 (48.18)	271.00	269.17 (88.15)	− 20.08	181.00	175.80 (46.60)	− 47.80	97.00	113.00 (63.79)	− 66.45
Indentation hardness (N/mm^2^)											
Distilled water	448.00	451.63 (59.70)	359.00	367.13 (65.95)	− 18.71	196.00	213.53 (65.36)	− 52.72	147.00	155.17 (28.86)	− 65.64
Chloramine-T	459.50	454.30 (54.50)	399.50	406.20 (77.49)	− 10.59	342.00	362.13 (86.55)	− 20.29	287.00	292.33 (73.55)	− 35.65
Thymol	455.50	444.07 (68.98)	326.00	352.90 (87.93)	− 20.53	250.00	247.90 (68.01)	− 44.18	212.00	210.13 (81.12)	− 52.68
Formalin	444.00	454.90 (66.77)	348.00	347.70 (123.40)	− 23.57	212.50	216.93 (58.38)	− 52.31	116.50	137.23 (79.42)	− 69.83
Indentation modulus (kN/mm^2^)											
Distilled water	11.65	11.07 (2.02)	10.55	10.38 (1.70)	− 6.23	7.55	7.23 (2.14)	− 34.64	5.00	5.15 (0.98)	− 53.46
Chloramine-T	11.15	11.38 (1.24)	11.50	11.60 (1.34)	+ 1.99	10.10	10.08 (1.57)	− 11.43	7.90	8.20 (1.25)	− 27.89
Thymol	10.70	10.54 (1.38)	9.85	9.83 (1.62)	− 6.70	8.20	7.90 (1.50)	− 25.08	6.20	6.11 (1.65)	− 42.00
Formalin	10.95	11.09 (1.67)	11.25	10.83 (2.44)	− 2.31	8.70	8.31 (2.14)	− 25.04	5.70	6.43 (3.12)	− 42.05
Indentation creep (%)											
Distilled water	5.50	5.52 (0.47)	5.80	5.98 (0.52)	+ 8.34	5.10	5.98 (1.74)	+ 8.34	5.10	5.45 (1.13)	− 1.17
Chloramine-T	5.50	5.51 (0.38)	5.90	5.80 (0.56)	+ 5.26	5.50	5.79 (0.90)	+ 5.08	5.25	5.35 (0.59)	− 2.90
Thymol	5.45	5.44 (0.49)	5.95	6.03 (0.64)	+ 10.91	5.50	5.72 (0.97)	+ 5.29	5.55	5.63 (0.52)	+ 3.49
Formalin	5.50	5.65 (0.68)	6.30	6.55 (1.02)	+ 15.92	7.30	7.39 (0.91)	+ 30.66	6.00	6.18 (1.11)	+ 9.38

Within 3 months of storage at the latest, all storage media resulted in significantly reduced values for *HM* and *H*_*IT*_ (both *p* < 0.001) with a percentage reduction of up to 49.75% and 52.72%, respectively. For all storage media, a reduction of *E*_*IT*_ was observed at 6 months at the latest (*p* < 0.001) with a percentage reduction of up to 53.46%. Regarding *C*_*IT*_, the formalin group showed significantly increased values from 1 month onward (*p* ≤ 0.042). In distilled water and thymol, a temporary increase after 1 month was found (both *p* ≤ 0.024).

Comparisons between the storage media at the same storage time showed significant differences at T3 and T6: Regarding *HM* and *H*_*IT*_, chloramine-T showed significantly higher values than distilled water, thymol, and formalin at T3 (*p*_adj._ ≤ 0.007) or than distilled water and formalin at T6 (*p*_adj._ ≤ 0.001). Regarding *E*_*IT*_, chloramine-T showed significantly higher values than distilled water at T3 and T6 (*p*_adj._ ≤ 0.011). Finally, *C*_*IT*_ was significantly increased in formalin compared to distilled water, chloramine-T, and thymol at T3 (*p*_adj._ ≤ 0.005).

## Discussion

In the present study, bovine teeth were chosen as a substitute for human teeth for several reasons: Firstly, the storage time can be substantially reduced while collecting the total number of teeth as bovine teeth are easily available. Secondly, caries and caries-associated complications are still the main reasons for tooth extraction, which compromises the acquisition of sound dental material^2,24^. Thirdly, as the mechanical properties of dentin change during lifetime, the confounding factor age can be remedied by using bovine teeth^2^. Moreover, bovine teeth show lower variability between samples, are more consistent in size and shape and are not affected by restoration procedures, which concludes to a higher comparability in testing samples^25^. The main reason for the usage of bovine teeth in the present study is the reduced time range needed to collect an adequate sample size while limiting the storage of the teeth prior to the experimental setup.

However, the microstructure of human and bovine teeth appears not to be identical. Several studies have investigated the similarity of bovine to human dentin regarding their physical properties and reported contradictory results^1,2,15,26–28^. Nevertheless, one study compared the Knoop hardness between bovine dentin from animals aged 20–48 months and human extracted teeth (aged 20–30 years) using indentation techniques^13^. In this regard, no differences between human and bovine dentin irrespective of the animals’ age was observed. Insofar, it can be assumed that the bovine specimens used in this study represent a homogeneous group almost comparable to human dentin aged 20–30 years.

Regarding the included sample size, only a few studies have previously investigated the mechanical properties of dentin, but with variations in the objective, testing procedure as well as storage media or time^4–7,13,20^. Consequently, these studies did not provide a suitable basis for an adequate power analysis. Therefore, the sample size in this study was determined based on investigations addressing a comparable objective and using indentation techniques^6,7,20^. Our results demonstrated that all storage media led to a highly significant reduction in Martens hardness after 3 months (*p* < 0.001), which was a frequently reported storage period prior to experimental use^29–32^. It is conceivable, however, that a larger sample size might have revealed significant effects at earlier time points. In addition, Xu et al.^32^ reported that a 20.8% reduction in the Knoop hardness of dentin was associated with a significant decrease in fracture strength, which can therefore be interpreted as a clinically relevant decline. While this percentage cannot be directly applied to our study, as we measured Martens rather than Knoop hardness, our results showed mean reductions between 18.74 and 49.75% after 3 months, predominantly exceeding 20.8%. Thus, beyond statistical significance, the percentage decrease in mean hardness values after 3 months also represents a relevant reduction, given the likely association with reduced fracture strength.

Indentation testing is a well-established method to examine dental materials and restorations. However, variations in hardness type (Martens, Vickers) and in setting parameters affect the results compromising comparison among publications^33^. To assess Martens hardness, measurements are conducted with instrument-guided indentation testing with continuous recording of the load and the indentation depth, independent of human perception or the resolution of optical equipment. To enable the comparison to previous studies, the generated force–displacement curve can be used to calculate Vickers hardness values^17,34,35^. Nevertheless, due to the consideration of the elastic component, Martens hardness is strongly recommended when evaluating materials presenting a specific amount of elastic recovery after loading^17^. Mean values of initial measurements for Martens hardness and indentation modulus in dentin were consistent with recently published values (*HM* = 200–800 N/mm^2^, *E*_*IT*_ = 5–25 kN/mm^2^, respectively, depending on the measured area within the root)^15,36^.

Compared to the Martens hardness which accounts for both plastic and elastic deformation, the indentation hardness solely relates to the resistance against plastic deformation^15,21^. Respectively higher values mean a stronger resistance potential of a material. When comparing Martens hardness and indentation hardness the latter tends to yield higher values, if the material exhibits elastic recovery or time-dependent deformation (indentation creep), as demonstrated in the results of this study^12^. The percentual changes in both parameters over the observation period show consistently declining results with a maximum difference of 4.51%. Corresponding to that, the indentation modulus relates to the elastic resistance of the material against deformation. While these values also showed reduced results over the storage time, the percentual reduction was higher in hardness parameters, changing the ratio to indentation modulus and therefore implying alternations in plasticity. More specifically, it indicates that the dentine lost hardness but is less prone to plastic deformation.

Consistent with the findings of the present study, previous research has shown that the storage of teeth generally results in a reduction of hardness and elastic modulus^6,7^. Moreover, it was clearly demonstrated that the storage duration had a significant impact on hardness alterations as a short-term storage of 7 days in thymol or distilled water did not influence the microhardness of dentin^20^. However, absolute values can hardly be compared directly, because storage media, duration, and hardness tests vary substantially^6,7,20^. Two studies indicated the percentage reduction of the Vickers hardness after storage in de-ionized water, which facilitates the comparison to the present study: Whereas Habelitz et al. stated a reduction of 25% after only 1 day storage time, Aydin et al. published a reduction of 35% after 10 months^6,7^. In the present study, storage in distilled water resulted in a percentage reduction of the Martens hardness of 16.3% and 63.4% after 4 weeks and 6 months, respectively. Considering storage time, the present reduction values appear to be within the range of previously published data.

Even though long-term storage affects the mechanical properties of dentin, standardization with regard to storage conditions of extracted teeth is missing^5^. Furthermore, not only the storage medium but also the necessity for regular replenishment of the solution is unclear. For the purpose of comparison with the existing literature all media were not replenished during the 6 months of storage^5,6^. Moreover, regular exchange of the liquid will provide a constant concentration gradient leading to further dissolution of inorganic ions. Therefore, keeping the storage solution will probably result in saturation of ion exchange. However, even without replenishment of the solution, the present study demonstrated a significant impact of the medium on the mechanical properties of dentine.

In detail, all storage media resulted in a significant decrease in Martens hardness within the first 3 months, with chloramine-T having the lowest impact. Except of distilled water, the tested solutions are known to have antibacterial efficacy and are commonly used as storage media^2,5,37^. Especially formalin and thymol solutions are widely used^6,18–20^.

Formalin has the potential to interfere with inorganic and organic components of dentin^8^. Especially unbuffered acidic formalin solutions dissolve calcium, phosphate, magnesium, and other components of hydroxyapatite, which increases porosity by reducing the mineral content. Moreover, storage in formalin solution results in an increased number of cross-linking due to the reaction of aldehydes with primary amine groups of collagen chains and may, therefore, alter the mechanical properties of dentin^38^. Which of these mechanisms has the higher impact on the reduction of hardness values is not clear and can only be assumed. Chloramine-T (sodium-*N*-Chlorotosylamide, pH 8–10) can denature collagen proteins in dentin and retains in the hard tissues even after rinsing or storing in distilled water for a prolonged period. Nevertheless, the extent and significance of this process remain unknown^25,37^. However, for both chemicals, provided that their concentration in aqueous solutions is sufficiently high, these reactions can occur to a measurable extent. Overall, current research on the impact of formalin and chloramine-T on dentine does not provide definitive explanations of the chemical interactions between these fluids and the organic or inorganic components of dentine. Thus, a causal relationship between the chemical potential and the negative impact on Martens parameters can only be assumed. Although chloramine-T showed the lowest effect on hardness parameters in the present study, it was previously recommended to refrain from using chloramine-T^25^. However, this recommendation was based on studies analyzing the discoloration of teeth. Therefore, the choice of the most suitable storage solution should be determined by the specific requirements of the specimens’ intended use.

The present and previous studies showed that even deionized or distilled water adversely affects the mechanical properties of dentin^6,7^. This phenomenon can be explained by the lack of ionized particles in the solution causing demineralization of the mineral phase of the dentin^7^. Moreover, not only storage in distilled water but also in formalin and thymol solutions resulted in mineral loss of dentin. The penetration depth into dentin clearly depends on storage media and time, with a maximum of up to 200 µm per week^4,39^. Nevertheless, the wash-out of the mineral phase seems to be limited to a thin superficial layer^4^. Therefore, it might be relevant to examine the influence of storage solutions in relation to the infiltration depth of the applied liquids.

## Conclusion

Depending on the storage medium, Martens parameters of bovine dentin can already be compromised after a storage time of 4 weeks. The choice of storage medium and time should be based on the intended use of the specimens. Within the limitations of this study using bovine dentin, it is reasonable to assume that the effect of storage media and time on the mechanical properties can be transferred to extracted human teeth.

## Supplementary Information

Below is the link to the electronic supplementary material.

Supplementary Material 1

## Data Availability

The datasets generated and analyzed during the current study are available from the corresponding author on reasonable request.
