# Supplementary material for: Impact of various storage media and time on mechanical properties of bovine root dentin
Source: Sci Rep. 2026 Apr 11;16:12182. doi: 10.1038/s41598-026-47214-1 (PMC13076640; doi:10.1038/s41598-026-47214-1)
Supplement: Supplementary file 1 — Supplementary Material 1 [file 41598_2026_47214_MOESM1_ESM.pdf]

<u>**Appendix**</u>

Supplemental Table S1: Mixed linear regression model (Martens hardness)

**Parameter**	**Martens hardness (N/mm^2), mean ± SD**	**Effect estimate, N/mm^2**	**Effect estimate, 95% confidence interval**	**p-value**
Distilled water *(intercept)*	333.73 ± 43.62	333.72	309.65 to 357.78	**<0.001***
Chloramine-T	338.13 ± 37.02	4.40	-29.63 to 38.43	0.801
Thymol	326.30 ± 45.97	-7.41	-41.44 to 26.62	0.672
Formalin	336.80 ± 48.18	3.09	-30.94 to 37.12	0.860
Distilled water : T1	279.30 ± 46.59	-45.32	-80.03 to -10.60	**0.017***
Chloramine-T : T1	310.20 ± 53.18	-17.95	-52.76 to 16.86	0.322
Thymol : T1	267.17 ± 59.51	-63.29	-97.92 to -28.66	**0.001***
Formalin : T1	269.17 ± 88.15	-70.80	-105.58 to -36.02	**<0.001***
Distilled water : T3	167.70 ± 50.42	-164.44	-197.96 to -130.92	**<0.001***
Chloramine-T : T3	274.77 ± 59.82	-61.62	-95.14 to -28.10	**<0.001***
Thymol : T3	192.72 ± 49.34	-137.11	-170.68 to -103.54	**<0.001***
Formalin : T3	175.80 ± 46.60	-161.55	-195.08 to -128.03	**<0.001***
Distilled water : T6	121.50 ± 27.93	-210.09	-248.39 to -171.80	**<0.001***
Chloramine-T : T6	222.07 ± 50.73	-113.72	-152.02 to -75.42	**<0.001***
Thymol : T6	160.80 ± 58.77	-166.48	-204.77 to -128.18	**<0.001***
Formalin : T6	113.00 ± 63.79	-224.54	-262.84 to -186.24	**<0.001***

T1: storage period of 1 month, T3: storage period of 3 months, T6: storage period of 6 months.

Supplemental Table S2: Mixed linear regression model (indentation hardness)

**Parameter**	**Indentation hardness (N/mm^2), mean ± SD**	**Effect estimate, N/mm^2**	**Effect estimate, 95% confidence interval**	**p-value**
Distilled water *(intercept)*	451.63 ± 59.70	451.47	417.82 to 485.13	**<0.001***
Chloramine-T	454.30 ± 54.50	2.63	-44.97 to 50.22	0.914
Thymol	444.07 ± 68.98	-7.34	-54.93 to 40.26	0.764
Formalin	454.90 ± 66.77	3.46	-44.14 to 51.05	0.888
Distilled water : T1	367.13 ± 65.95	-71.37	-121.23 to -21.50	**0.008***
Chloramine-T : T1	406.20 ± 77.49	-31.65	-81.67 to 18.36	0.223
Thymol : T1	352.90 ± 87.93	-97.06	-146.80 to -47.33	**<0.001***
Formalin : T1	347.70 ± 123.40	-109.64	-159.62 to -59.67	**<0.001***
Distilled water : T3	213.53 ± 65.36	-234.56	-278.99 to -190.12	**<0.001***
Chloramine-T : T3	362.13 ± 86.55	-87.73	-132.18 to -43.29	**<0.001***
Thymol : T3	247.90 ± 68.01	-201.55	-246.06 to -157.05	**<0.001***
Formalin : T3	216.93 ± 58.38	-238.63	-283.07 to -194.18	**<0.001***
Distilled water : T6	155.17 ± 38.86	-293.13	-344.86 to -241.39	**<0.001***
Chloramine-T : T6	292.33 ± 73.55	-157.78	-209.53 to -106.04	**<0.001***
Thymol : T6	210.13 ± 81.12	-235.43	-287.16 to -183.71	**<0.001***
Formalin : T6	137.23 ± 79.42	-318.29	-370.03 to -266.55	**<0.001***

T1: storage period of 1 month, T3: storage period of 3 months, T6: storage period of 6 months.

Supplemental Table S3: Mixed linear regression model (indentation modulus)

Parameter	Indentation modulus (kN/mm^2), mean ± SD	Effect estimate, kN/mm^2	Effect estimate, 95% confidence interval	p-value
Distilled water *(intercept)*	11.07 ± 2.02	11.03	10.05 to 12.02	**<0.001***
Chloramine-T	11.38 ± 1.24	0.33	-1.06 to 1.72	0.648
Thymol	10.54 ± 1.38	-0.48	-1.87 to 0.91	0.506
Formalin	11.09 ± 1.67	0.08	-1.31 to 1.47	0.907
Distilled water : T1	10.38 ± 1.70	-0.46	-1.36 to 0.44	0.324
Chloramine-T : T1	11.60 ± 1.34	0.34	-0.57 to 1.25	0.466
Thymol : T1	9.83 ± 1.62	-0.83	-1.73 to 0.07	0.080
Formalin : T1	10.83 ± 2.44	-0.45	-1.36 to 0.46	0.337
Distilled water : T3	7.23 ± 2.14	-3.90	-5.27 to -2.53	**<0.001***
Chloramine-T : T3	10.08 ± 1.57	-1.33	-2.70 to 0.04	0.065
Thymol : T3	7.90 ± 1.50	-2.69	-4.06 to -1.32	**<0.001***
Formalin : T3	8.31 ± 2.14	-2.72	-4.09 to -1.35	**<0.001***
Distilled water : T6	5.15 ± 0.98	-5.91	-7.31 to -4.51	**<0.001***
Chloramine-T : T6	8.20 ± 1.25	-3.17	-4.57 to -1.77	**<0.001***
Thymol : T6	6.11 ± 1.65	-4.43	-5.84 to -3.03	**<0.001***
Formalin : T6	6.43 ± 3.12	-4.67	-6.07 to -3.27	**<0.001***

T1: storage period of 1 month, T3: storage eriod of 3 months, T6: storage period of 6 months.

Supplemental Table S4: Mixed linear regression model (indentation creep)

Parameter	Indentation creep (%), mean ± SD	Effect estimate, %	Effect estimate, 95% confidence interval	p-value
Distilled water *(intercept)*	5.52 ± 0.47	5.51	5.26 to 5.77	**<0.001***
Chloramine-T	5.51 ± 0.38	0.01	-0.32 to 0.35	0.937
Thymol	5.44 ± 0.49	-0.07	-0.42 to 0.27	0.677
Formalin	5.65 ± 0.68	0.15	-0.20 to 0.50	0.401
Distilled water : T1	5.98 ± 0.52	0.46	0.08 to 0.84	**0.024***
Chloramine-T : T1	5.80 ± 0.56	0.26	-0.13 to 0.64	0.197
Thymol : T1	6.03 ± 0.64	0.58	0.20 to 0.96	**0.005***
Formalin : T1	6.55 ± 1.02	0.89	0.50 to 1.27	**<0.001***
Distilled water : T3	5.98 ± 1.74	0.43	-0.28 to 1.15	0.240
Chloramine-T : T3	5.79 ± 0.90	0.15	-0.57 to 0.87	0.682
Thymol : T3	5.72 ± 0.97	0.18	-0.53 to 0.89	0.620
Formalin : T3	7.39 ± 0.91	1.73	1.02 to 2.45	**<0.001***
Distilled water : T6	5.45 ± 1.13	0.00	-0.50 to 0.49	0.994
Chloramine-T : T6	5.35 ± 0.59	-0.19	-0.67 to 0.29	0.441
Thymol : T6	5.63 ± 0.52	0.18	-0.30 to 0.66	0.475
Formalin : T6	6.18 ± 1.11	0.52	0.04 to 1.00	**0.042***

T1: storage period of 1 month, T3: storage period of 3 months, T6: storage period of 6 months.
